# Supplementary material for: Synergistic olfactory processing for social plasticity in desert locusts
Source: Nat Commun. 2024 Jun 28;15:5476. doi: 10.1038/s41467-024-49719-7 (PMC11213921; doi:10.1038/s41467-024-49719-7)
Supplement: Supplementary file 1 — Supplementary Information [file 41467_2024_49719_MOESM1_ESM.pdf]

Supplementary information:

Synergistic olfactory processing for social plasticity in desert locusts

Inga Petelski[a,b,c,+], **Yannick Günzel**[a,b,c,d,+,*], **Sercan Sayin**[b,d], **Susanne Kraus**[b], **and Einat Couzin-Fuchs**[b,c,d,*]

[a]International Max Planck Research School for Quantitative Behavior, Ecology and Evolution from lab to field, 78464 Konstanz, Germany
[b]Department of Biology, University of Konstanz, 78464 Konstanz, Germany
[c]Department of Collective Behavior, Max Planck Institute of Animal Behavior, 78464 Konstanz, Germany
[d]Centre for the Advanced Study of Collective Behaviour, University of Konstanz, 78464 Konstanz, Germany
[+]contributed equally
[*]To whom correspondence should be addressed. E-mail: yannick.guenzel@uni-konstanz.de (ORCID: 0000-0001-7553-4742), einat.couzin@uni-konstanz.de (ORCID: 0000-0001-5269-345X)

Content

This document contains:

- Supporting methods
- Supporting figures 01 - 06
- Supporting tables 01 - 02

1 Supporting methods

1.1 Widefield calcium imaging

We conducted calcium imaging analysis at a widefield fluorescence microscope (BX51WI, Olympus, Tokyo, Japan), equipped with a 20x water immersion objective (Olympus, UM Plan Fl 20x/0.50W), using an Omicron LED system, equipped with a 470 nm LED (Omicron-Laserage Laserprodukte GmbH, Rodgau-Dudenhofen, Germany) for light excitation with a 410 nm short-pass filter and a 410 nm dichroic mirror. In the emission pathway, the light was filtered through a 440 nm long-pass filter. Images with a spatial resolution of approx. 1.59 px/μm were captured at 10 fps with a sCMOS camera (1024x1024 pixel with 2x2 on-chip binning; Prime BSI Express; Teledyne Photometrics, Tuscon AZ, USA).

Odors were delivered via an automatic multi-sampler for gas chromatography (Combi PAL, CTC Analytics AG, Zwingen, Switzerland), which injected the odor pulse into a continuous flow of 1 mL/s of purified air with a matching injection speed. The stimulus odor was directed via a Teflon tube (inner diameter, 0.87 mm) to the antenna ipsilateral to the imaging site. Leaf extract (*Lvs*) was produced by grinding blackberry leaves in mineral oil. Odor vials were filled with either 0.5 g of colony cotton wool, 0.3 g leaf extract, or 200 μL of cis-3-Hexenyl-acetate (diluted to 10^{-2}, *Laa*) on 0.5 g of pure cotton wool. Mixtures were prepared by combining half the amount of the respective odor dilution, leaf extract, or colony cotton wool.

Data preprocessing followed the same steps as described in the main Methods section, with the only differences in the median filter size (11-by-11 pixels) for spatial filtering, resizing the images to 256x256 pixels, the box-shaped kernel size (11 frames) for temporal filtering, and baseline length (20 frames) for calculating relative fluorescence changes ($\Delta F/F_0 = \frac{F-F_0}{F_0}$).

1.2 Electroantennography (EAG) recordings

We conducted EAG recordings to measure the voltage fluctuations between the tip and the base of antennae from gregarious and solitarious animals, elicited in response to a social olfactory stimulus (the smell of the colony, *Lct*), a food-related stimulus (leaf alcohol acetate, *Laa*), and their mixture (*LaaLct*; same stimuli and their preparation as described in the main text for functional confocal microscopy). In preparation for the EAG experiments, we inserted the base and the tip of a freshly dissected antenna into glass capillaries (1.2 mm OD x 0.69 mm ID, Harvard Apparatus, Holliston, MA, United States) filled with locust saline (9.82 g/L $NaCl$; 0.48 g/L KCl; 0.73 g/L $MgCl_2 * 6H_20$; 0.47 g/L $CaCl_2 * 6H_2O$; 0.95 g/L $NaH_2P0_4 * 2H_2O$; 0.18 g/L $NaHCO_3$; pH of 7.2 with $NaOH$; after: (1)), connected to electrode holders. The tip electrode and base electrode were connected to an Axon Instruments CV-7B headstage, and voltage deflections were amplified using a MultiClamp 700B Microelectrode Amplifier (Molecular Devices, Sunnyvale, CA, United States) with respective MultiClamp 700B Commander software. We recorded the data at 25 KHz with a Micro1401 A/D converter and Spike2 data acquisition software (v7.01, Cambridge Electronic Design, Cambridge, UK), as illustrated in suppl. Fig. 3a. Baseline drift was accounted for by online or offline high-pass filtering (cutoff frequency of 3 Hz) followed by offline low-pass filtering (100 Hz) of the signal (suppl. Fig. 3b).

Using the same custom-built olfactometer as for the functional confocal microscopy experiments (see main text for details; (2)), we delivered each stimulus as three consecutive, 2-second-long odor pulses with an inter-pulse interval of 10 s. Stimuli were presented in a pseudo-random.

For each of the three repetitions, we used two-second long pre- and post-stimulus intervals and normalized the data to the minimum (i.e., most extreme) value in the respective response to *Laa* (e.g., first repetition to *Lct* was normalized with the first repetition to *Laa*). Next, we averaged the repetitions to the same stimulus before normalizing to *Laa* again to account for subtle differences between repetitions. Consequently, the relative response magnitude was measured as the minimum value during stimulus presentation, with values less negative than -1 indicating weaker responses and values more negative than -1 indicating stronger responses compared to *Laa*.

For statistical inference, we used bootstrap randomization tests with $B = 10^7$ samples and a one-sample, two-tailed test statistic (suppl. Eqn. 1). To test a sample with mean $\bar{z}$, standard deviation σ_z, and sample size n against a pre-determined value μ_0 (-1 in our case) we drew samples from the empirical distribution $\tilde{z}_i = z_i - \bar{z} + \mu_0$ with $i = 1, \ldots, n$ (see main text for details on bootstrap randomization tests). We used the same test statistic as denoted in the main text for unpaired, two-sample, two-tailed tests, comparing gregarious with solitarious data (Eqn. 5).

$$T_{one-sample} = \frac{|\bar{z} - \mu_0|}{\sigma_z / n} \quad (1)$$

1.3 Temporal evolution of odor responses in principal component space

Projecting response patterns of neuronal ensembles into a common space can offer more insight into the evolution of temporal patterns. To this end, we concatenated the data of all cell bodies from all gregarious and solitarious animals to project them into principal component (PC) space (based on singular value decomposition), obtaining a single coefficient and a single score matrix for our dataset. Using the matching columns of the coefficient matrix, we then projected each animal's data into PC-space. This allowed us to calculate the grand mean (mean of animal means) trajectories for gregarious and solitarious animals, respectively. Moreover, we used the score matrix to estimate the effect of the first PC. For this, we projected the data back: once using the full coefficient and score matrices, and once excluding the first principal component.

2 Supporting figures

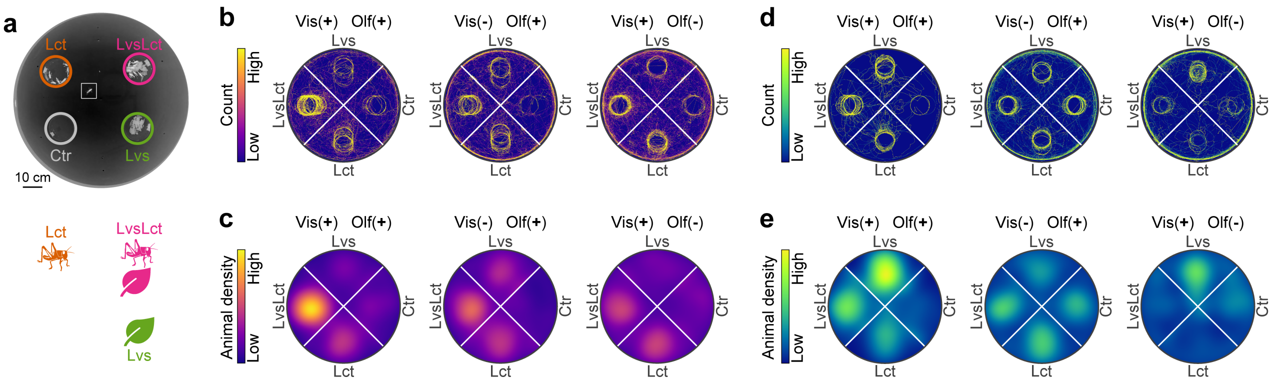

Supplementary figure 1: Details on patch selection experiments.
a Animals were tested in a circular arena with four patch options (blackberry leaves Lvs, locusts Lct, leaves and locusts $LvsLct$, and control Ctr) under one out of three conditions (visual and olfactory cues available: $Vis(+)Olf(+)$; olfactory but no visual cues: $Vis(-)Olf(+)$; visual but no olfactory cues: $Vis(+)Olf(-)$). Shown is a color-inverted, exemplary frame from testing a gregarious animal. The superimposed circles indicate the four patches, and the square shows the location of the locust. The animal was tested with both visual and olfactory cues available. **b** Bivariate histograms with 1000-by-1000 equally spaced bins were used to pool the 2-D trajectories of all tested gregarious animals ($Vis(+)Olf(+)$: $n = 26$; $Vis(-)Olf(+)$: $n = 26$; $Vis(+)Olf(-)$: $n = 28$). **c** Bivariate histograms from b were used for estimating animal density in each stimulus quarter (same as in Fig. 1). **d-e** As in b-c, but for solitarious animals ($Vis(+)Olf(+)$: $n = 25$; $Vis(-)Olf(+)$: $n = 32$; $Vis(+)Olf(-)$: $n = 25$).

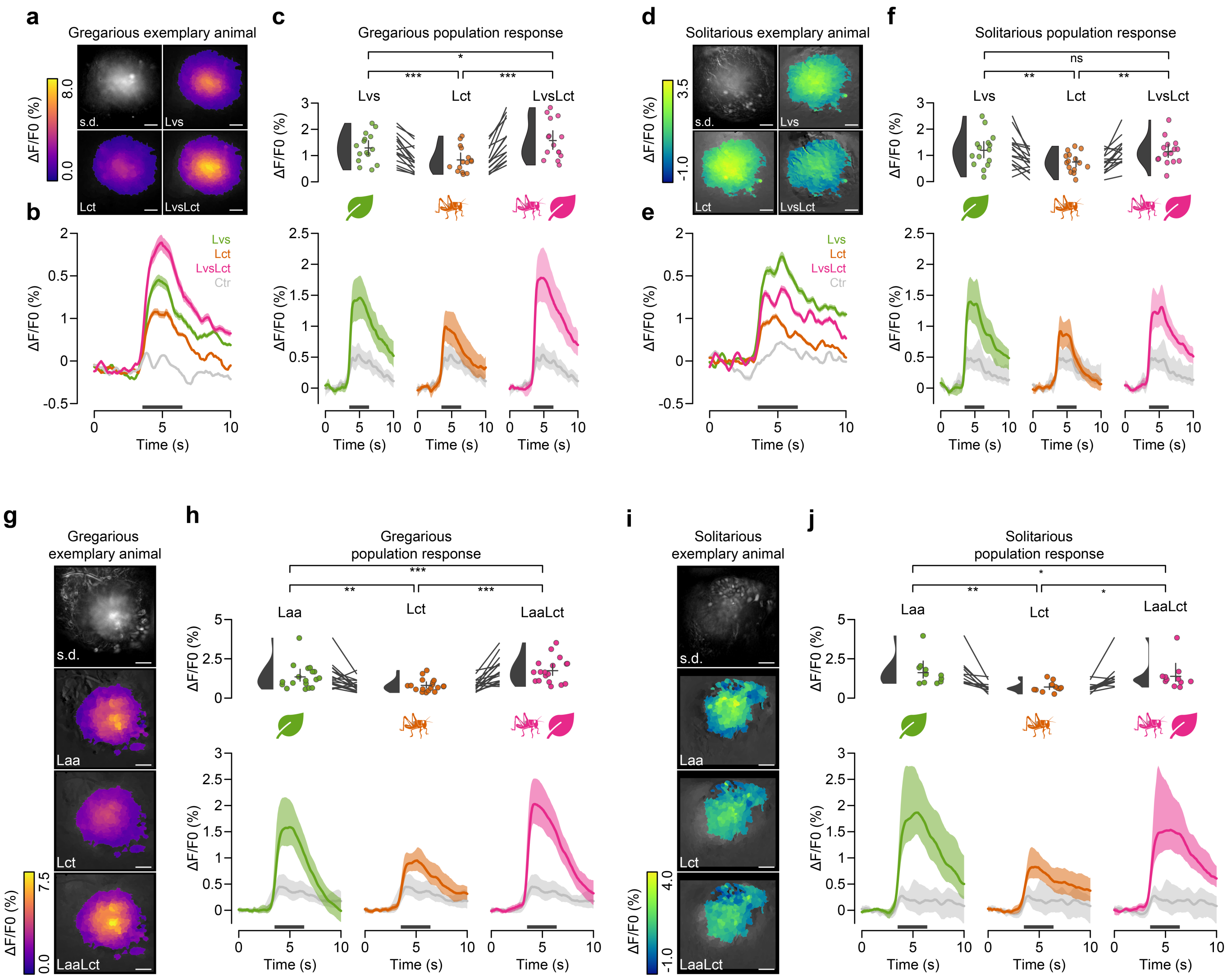

Supplementary figure 2: Functional widefield imaging of the antennal lobe representation of the odor cues used in patch choice experiments.

a Widefield calcium imaging data following the presentation of the olfactory stimuli used in the patch selection assay (blackberry leave extract: *Lvs*, locust odor *Lct*, and their combination *LvsLct*). The grayscale heatmap shows the standard deviation projection across all stimuli of an exemplary gregarious animal, alongside the mean intensity projection after olfactory stimulation during the window of activity (see b). **b** Average activity time courses (mean across all active regions with shaded 95% confidence intervals) of the exemplary animal shown in a for each stimulus (air control in light gray). **c** Average activity time courses of all $n = 15$ gregarious animals (grand means with shaded 95% confidence intervals, bottom panels) and respective swarm plots of the individual animal means during the window of activity (dark gray bars) above, with half-violin plots for probability density estimates, and crosses for grand means and 95% confidence intervals ($p_{(Lvs/LvsLct)} = 0.0217$, $p_{(Lvs/Lct)} = 1.7022 * 10^{-4}$, $p_{(Lct/LvsLct)} = 8.28 * 10^{-6}$). **d-f** Same as a-c, but for $n = 15$ solitarious animals with $p_{(Lvs/LvsLct)} > 0.99$, $p_{(Lvs/Lct)} = 0.0067$, and $p_{(Lct/LvsLct)} = 0.0093$. **g** Same as in a, but for leaf alcohol acetate (*Laa*, cis-3-Hexenyl Acetate) instead of *Lvs*, as *Laa* could be identified as a dominant volatile in blackberry leaves (cf. Fig. 2e). **h** Same as in c, but for leaf alcohol acetate and $n = 18$ gregarious animals ($p_{(Laa/LaaLct)} = 5.481 * 10^{-4}$, $p_{(Laa/Lct)} = 0.0094$, $p_{(Lct/LaaLct)} = 3.774 * 10^{-5}$). **i-j** Same as g-h, but for $n = 11$ solitarious animals ($p_{(Laa/LaaLct)} = 0.0284$, $p_{(Laa/Lct)} = 0.0027$, $p_{(Lct/LaaLct)} = 0.0431$). Statistical inference in c, f, h, and j was based on paired two-sample two-tailed bootstrap randomization tests with Bonferroni correction accounting for multiple comparisons (ns: not significant; $p < 0.05$: *; $p < 0.01$: **; $p < 0.001$: ***). Source data are provided as a Source Data file.

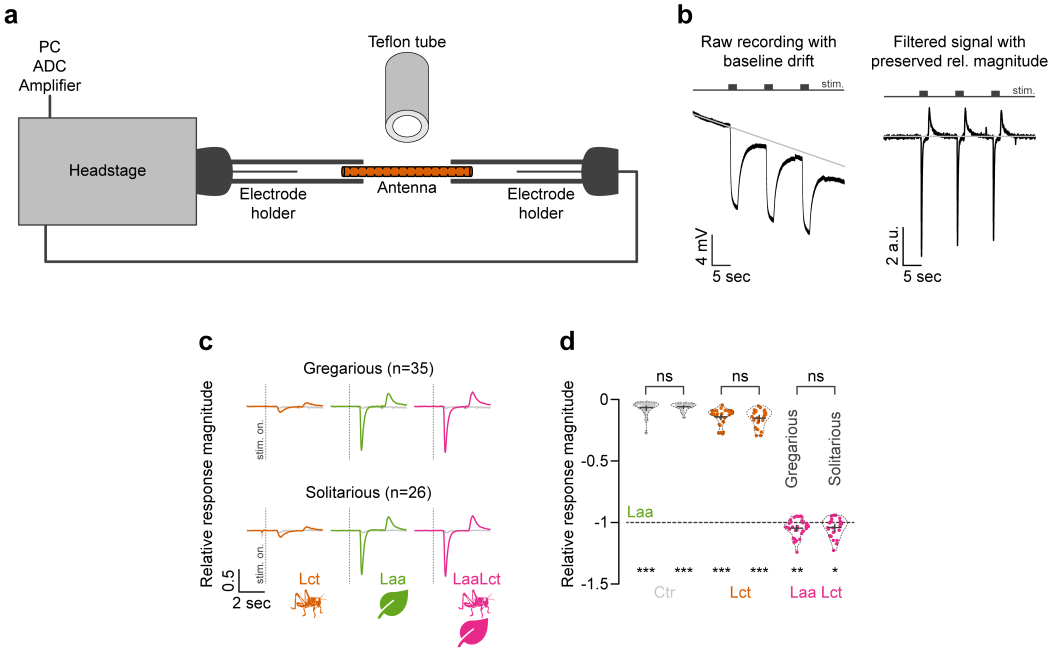

Supplementary figure 3: Electroantennogram (EAG) analysis of the odor cues used in patch choice experiments. **a** Schematic illustration of the EAG setup used to record voltage fluctuations along a locust antenna. **b** EAG recordings can be affected by drifting baselines that may complicate an exact calculation of response magnitudes. For this reason, we filtered the signal (3 Hz and 100 Hz cutoff frequencies for high-pass and low-pass filtering, respectively), preserving the relative differences between responses while accounting for the drift. This is exemplified by a raw recording from a gregarious antenna. **c** Average activity time courses of all gregarious (top row; $n = 35$) and solitarious (bottom row; $n = 26$) antennae in response to stimulation with leaf alcohol acetate *Laa*, the locust odor *Lct*, and their combination *LaaLct*. Responses to the solvent are shown as light gray time courses. Stimulus onset is indicated by vertical dotted lines. **d** Response magnitudes for all antennae are shown as swarm plots (pairwise for each stimulus, with gregarious on the left and solitarious on the right; same color code as in c). Responses were normalized to *Laa* (dotted horizontal line). Statistical inference in d was based on one-sample two-tailed bootstrap randomization tests to test whether values are different from *Laa*, and unpaired two-sample two-tailed bootstrap randomization tests to compare gregarious with solitarious responses (ns: not significant; $p < 0.05$: *; $p < 0.01$: **; $p < 0.001$: *** with $p_{(Ctr)} = 0.5399$, $p_{(Lct)} = 0.5219$, and $p_{(LaaLct)} = 0.7656$). Source data are provided as a Source Data file.

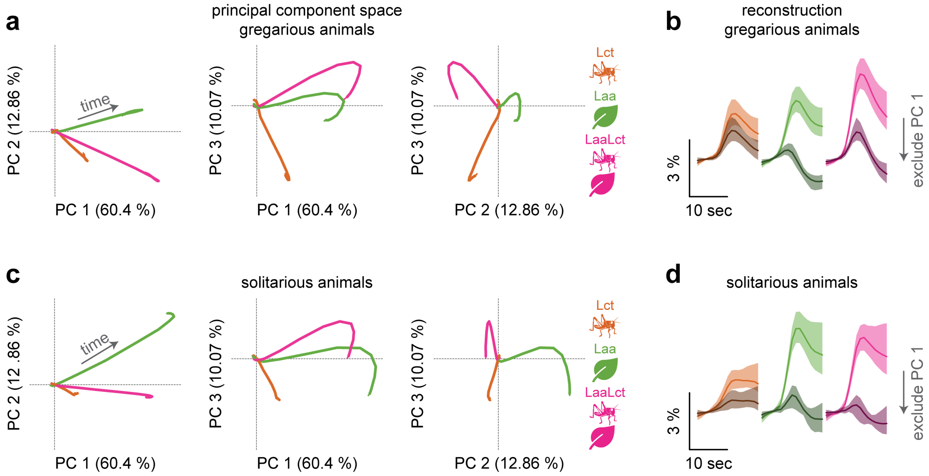

Supplementary figure 4: Principal component trajectories of temporally evolving odor response patterns differ between stimuli.

a Trajectories of gregarious (top row; $n = 28$) and solitarious (bottom row; $n = 19$) animals, projected into principal component space, of odor-induced responses in projection neuron cell bodies to the locust odor *Lct* (orange), leaf alcohol acetate *Laa* (green), and their combination *LaaLct* (magenta). **b** Reconstruction of the original signals, either based on all principal components (light shades) or all but the first (dark shades). **c-d** Same as a-b, but for solitarious animals. Source data are provided as a Source Data file.

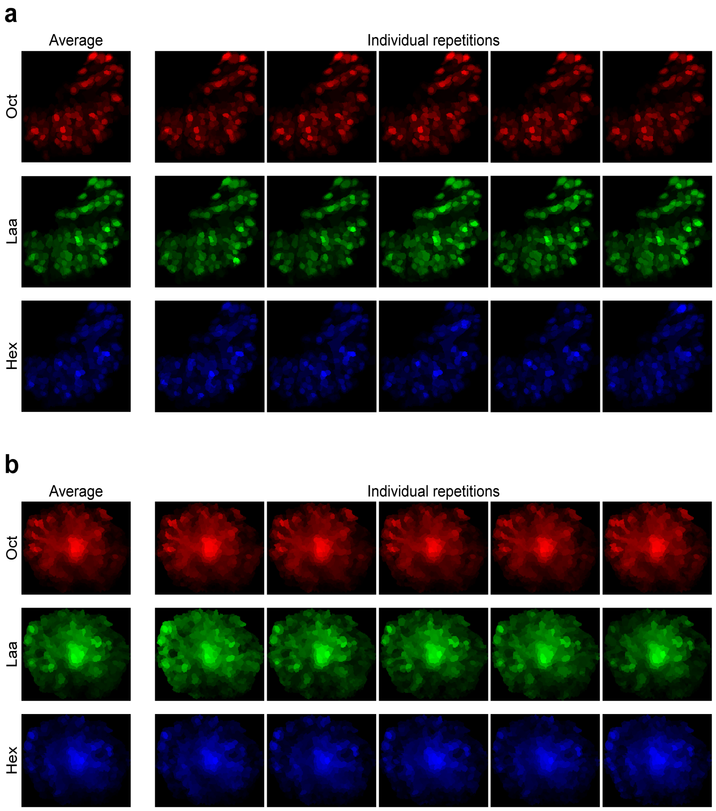

Supplementary figure 5: Details on response consistencies.
Individual trials of the example animals in Fig. 4a-b. Shown are odor-based color-coded standard deviation intensity projections of projection neuron cell bodies (**a**) and glomeruli (**b**) to repeated presentations of the odorants 1-Octanol *Oct*, leaf alcohol acetate *Laa*, and 1-Hexanol *Hex*. The left panels show average responses (as in Fig. 4) with the individual repetitions beside them.

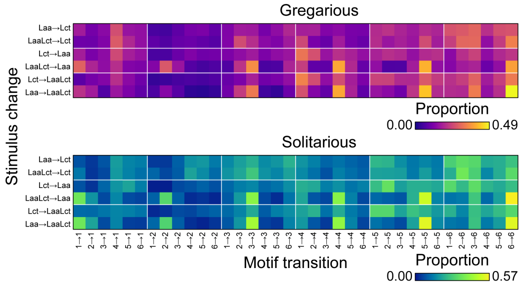

Supplementary figure 6: Response motif transition probabilities. Visualization of the transition probabilities between response motifs used for phenotype classifications in Fig. 5. Color codes for the proportion of cell bodies undergoing transitions between motifs (columns) across two stimulations (rows). Data for gregarious animals are in the top panel, with solitarious response transition probabilities below.

3 Supporting tables

Sets	cell bodies (greg.)	cell bodies (soli.)	glomeruli (greg.)	glomeruli (soli.)
Laa only	0.07 [0.05,0.11]	0.09 [0.07,0.13]	0.06 [0.04,0.11]	0.23 [0.11,0.48]
Lct only	0.13 [0.11,0.15]	0.15 [0.12,0.19]	0.08 [0.04,0.15]	0.14 [0.08,0.23]
LaaLct only	0.11 [0.08,0.15]	0.06 [0.04,0.12]	0.12 [0.08,0.18]	0.05 [0.02,0.08]
Laa & Lct	0.06 [0.05,0.09]	0.06 [0.05,0.08]	0.03 [0.02,0.06]	0.04 [0.02,0.05]
Laa & LaaLct	0.19 [0.16,0.23]	0.26 [0.22,0.31]	0.31 [0.20,0.42]	0.19 [0.13,0.26]
Lct & LaaLct	0.13 [0.11,0.15]	0.07 [0.05,0.09]	0.11 [0.06,0.22]	0.05 [0.02,0.10]
all	0.32 [0.27,0.40]	0.31 [0.26,0.35]	0.29 [0.22,0.38]	0.31 [0.21,0.39]

Supplementary table 1: Average proportion (grand mean [95% confidence interval]) of PNs responding in functional confocal laser scanning microscopy for the logical relationship between stimuli.

motif triplet	greg.	soli.	abs. diff.
2|5|5	1.49	6.23	4.74
1|4|4	6.17	2.67	3.51
1|1|6	3.00	0.38	2.62
1|1|4	3.23	0.80	2.43
6|3|3	3.12	5.34	2.22
3|6|3	1.31	3.47	2.16
2|4|4	2.10	4.07	1.97
2|4|5	0.96	2.92	1.96
3|6|6	5.01	6.86	1.85
6|4|5	2.97	1.23	1.74
2|6|6	1.49	3.22	1.73
6|6|3	1.51	3.18	1.66

Supplementary table 2: Details on the prevalence of triplet combinations. We calculated how many times more a given motif triplet occurred than chance level (gregarious: 34.33; solitarious: 23.61) for both locust phenotypes. We report the 12 triplet combinations with the largest absolute difference between gregarious and solitarious animals, matching the Bliss interaction score time courses in Fig. 5f. A value of 1 indicates that the triplet occurred as many times as expected by chance.

References

[1] Mordue, W. t. & Goldsworthy, G. The physiological effects of corpus cardiacum extracts in locusts. General and comparative Endocrinology **12**, 360–369 (1969).

[2] Raiser, G., Galizia, C. G. & Szyszka, P. A high-bandwidth dual-channel olfactory stimulator for studying temporal sensitivity of olfactory processing. Chemical senses **42**, 141–151 (2017).
